# Free-breathing radial magnetic resonance elastography of the liver in children at 3 T: a pilot study

**DOI:** 10.1007/s00247-022-05297-8

**Published:** 2022-04-02

**Authors:** Sevgi Gokce Kafali, Tess Armstrong, Shu-Fu Shih, Grace J. Kim, Joseph L. Holtrop, Robert S. Venick, Shahnaz Ghahremani, Bradley D. Bolster, Claudia M. Hillenbrand, Kara L. Calkins, Holden H. Wu

**Affiliations:** 1 Department of Radiological Sciences, David Geffen School of Medicine, University of California Los Angeles, 300 UCLA Medical Plaza, Suite B119, Los Angeles, CA 90095 USA; 2 Department of Bioengineering, University of California Los Angeles, Los Angeles, CA USA; 3 Department of Diagnostic Imaging, St. Jude Children’s Research Hospital, Memphis, TN USA; 4 Department of Pediatrics, David Geffen School of Medicine, University of California Los Angeles, Los Angeles, CA USA; 5 Siemens Medical Solutions, Inc., Salt Lake City, UT USA; 6 Research Imaging NSW, University of New South Wales, Sydney, Australia

**Keywords:** Children, Fibrosis, Free-breathing, Liver, Magnetic resonance elastography, Magnetic resonance imaging, Radial imaging, Stiffness

## Abstract

**Background:**

Magnetic resonance (MR) elastography of the liver measures hepatic stiffness, which correlates with the histopathological staging of liver fibrosis. Conventional Cartesian gradient-echo (GRE) MR elastography requires breath-holding, which is challenging for children. Non-Cartesian radial free-breathing MR elastography is a potential solution to this problem.

**Objective:**

To investigate radial free-breathing MR elastography for measuring hepatic stiffness in children.

**Materials and methods:**

In this prospective pilot study, 14 healthy children and 9 children with liver disease were scanned at 3 T using 2-D Cartesian GRE breath-hold MR elastography (22 s/slice) and 2-D radial GRE free-breathing MR elastography (163 s/slice). Each sequence was acquired twice. Agreement in the stiffness measurements was evaluated using Lin’s concordance correlation coefficient (CCC) and within-subject mean difference. The repeatability was assessed using the within-subject coefficient of variation and intraclass correlation coefficient (ICC).

**Results:**

Fourteen healthy children and seven children with liver disease completed the study. Median (±interquartile range) normalized measurable liver areas were 62.6% (±26.4%) and 44.1% (±39.6%) for scan 1, and 60.3% (±21.8%) and 43.9% (±44.2%) for scan 2, for Cartesian and radial techniques, respectively. Hepatic stiffness from the Cartesian and radial techniques had close agreement with CCC of 0.89 and 0.94, and mean difference of 0.03 kPa and −0.01 kPa, for scans 1 and 2. Cartesian and radial techniques achieved similar repeatability with within-subject coefficient of variation=1.9% and 3.4%, and ICC=0.93 and 0.92, respectively.

**Conclusion:**

In this pilot study, radial free-breathing MR elastography was repeatable and in agreement with Cartesian breath-hold MR elastography in children.

**Supplementary Information:**

The online version contains supplementary material available at 10.1007/s00247-022-05297-8.

## Introduction

Chronic liver disease in children is a major health problem [1]. In young children, biliary atresia, metabolic disease and idiopathic hepatitis are common causes of chronic liver disease [2]. In older children, autoimmune hepatitis, viruses, nonalcoholic fatty liver disease (NAFLD), cystic fibrosis and primary sclerosing cholangitis are common causes [3]. Chronic liver disease can lead to fibrosis and cirrhosis, which can cause portal hypertension, variceal bleeding, hepatocellular carcinoma, liver failure and even death [4, 5].

Accurate fibrosis staging is essential for clinical management and assessing prognosis [6]. The reference standard to diagnose and stage fibrosis is a liver biopsy. There are four fibrosis stages according to the METAVIR scoring system: F0: no fibrosis, F1: portal fibrosis without septa, F2: portal fibrosis with few septa, F3: numerous septa without cirrhosis, F4: cirrhosis [7]. Unfortunately, liver biopsies are limited by sampling error and low intra- and interobserver repeatability and agreement [8–10]. In children, biopsies often require anesthesia and are associated with complications [8, 9]. While ultrasound (US) is a noninvasive alternative to biopsy, the sensitivity of US is reduced at deep body regions, particularly in obese patients. As a result, the area under the receiver operator curve (AUC) is low (e.g., 0.67) for hepatic fibrosis detection using US [11].

Magnetic resonance (MR) elastography is a safe, noninvasive imaging modality that accurately and repeatably [12, 13] measures hepatic stiffness (in kPa), which correlates with liver biopsy results in adults [9] and children [14]. Compared to biopsy, MR elastography provides spatial maps of hepatic stiffness and overcomes the sampling limitations of biopsy, especially when there is heterogeneity of hepatic stiffness in different liver segments [15]. MR elastography is typically performed using 2-D Cartesian gradient-echo (GRE) or spin-echo echo-planar imaging (SE-EPI)-based pulse sequences to acquire 2 to 4 slices covering the mid-liver [16, 17]. Cartesian MR elastography scans are usually acquired during breath-holding to avoid motion artifacts. In adults, when compared to liver biopsy, Cartesian GRE breath-hold MR elastography differentiates F0 versus F1-F4 with sensitivity and specificity of 98% and 99%, respectively [18].

In children, Cartesian GRE breath-hold MR elastography has a lower sensitivity and specificity for detecting hepatic fibrosis compared to the performance in adults. One study found Cartesian GRE breath-hold MR elastography to have a sensitivity of 44–47% and specificity of 89–91% for differentiating F0 versus F1-F4 in children [19]. Another study reported a sensitivity of 88% and specificity of 85% for detecting F2-F4 [20]. These results may be because children are often restless during the exam and cannot successfully breath-hold and follow instructions [19, 21–23]. For these reasons, some studies exclude technical failure cases. Technical failure rates vary from 4% [24] to 16% [19] for breath-hold MR elastography in children.

To overcome the challenges associated with breath-holding, a few studies in adults investigated the feasibility of Cartesian GRE free-breathing MR elastography and its agreement with conventional Cartesian GRE breath-hold MR elastography. While one study of Cartesian GRE free-breathing MR elastography showed excellent agreement with Cartesian GRE breath-hold MR elastography for quantifying hepatic stiffness [25], another study showed low agreement [26]. Studies in children with Cartesian GRE free-breathing MR elastography are limited [8]. In contrast to Cartesian sampling, non-Cartesian radial sampling has greater inherent robustness to motion artifacts and offers advantages for free-breathing acquisition. In children, a multi-echo GRE free-breathing MRI sequence based on a 3-D stack-of-stars radial trajectory has been shown to achieve accurate and repeatable quantification of hepatic proton-density fat fraction, a biomarker for steatosis, and R_2_*, a biomarker for iron deposition, due to its inherent motion robustness [27, 28]. In a small study that included four subjects with ages ranging from 14 to 52 years, a 2-D radial GRE free-breathing MR elastography sequence demonstrated encouraging feasibility at 1.5 T [29]. Further research is needed to investigate the performance of 2-D radial GRE free-breathing MR elastography in children.

Hence, in this pilot study of healthy children and children with chronic liver disease, we aimed to characterize the technical performance of 2-D radial GRE free-breathing MR elastography with respect to 2-D Cartesian GRE breath-hold MR elastography. We sought to determine: 1) the measurable liver area on hepatic stiffness maps, 2) agreement in measuring hepatic stiffness and 3) intra-session repeatability for measuring hepatic stiffness.

## Materials and methods

### Study population and design

The inclusion criteria for the healthy cohort were 6–17 years of age and the ability to perform breath-holds (approximately 20 s). The inclusion criteria for the liver disease cohort were 6–17 years of age, the ability to perform breath-holds (approximately 20 s) and chronic liver disease such as NAFLD, nonalcoholic steatohepatitis, intestinal failure associated liver disease, viral/medication-induced hepatitis, autoimmune sclerosing cholangitis, Wilson disease, hemosiderosis, acute/chronic liver rejection, metabolic/genetic disorders affecting the liver, biliary atresia, idiopathic liver disease or any other chronic liver disease known to cause fibrosis. We opted to enroll children >6 years of age since most children in this age range can breath-hold and follow instructions. Exclusion criteria for the healthy control cohort included body mass index (BMI) >85th percentile. For the children with chronic liver disease, exclusion criteria included infections known to affect the liver. For both cohorts, we excluded children with congenital malformations of the liver, inborn error of metabolism, contraindications to MRI (claustrophobia, metallic objects in the body) and pregnancy.

One subject had a clinical MRI and MR elastography exam followed by a 30-min add-on research MR elastography protocol. All the remaining subjects were scanned with a 90-min research-only MRI and MR elastography protocol. All subjects were scanned after nothing by mouth for 3–4 h [30]. Data were entered into a secure database for management and analysis [31].

### MR elastography experiments

Before the start of the exam, the study coordinators showed videos and pictures of the MRI scanner to each subject. Additionally, the MR technologists explained the effects of the vibration of the MR elastography paddle before the experiments. Experiments were performed on a 3-T MRI scanner (MAGNETOM PrismaFit; Siemens Healthcare GmbH, Erlangen, Germany) with an MR elastography system (Resoundant, Rochester, MN), using the 18-channel body array and 32-channel spine array coils. Subjects were scanned using a 2-D Cartesian GRE breath-hold MR elastography (Cartesian breath-hold MR elastography) sequence and a prototype golden angle-ordered 2-D radial GRE free-breathing MR elastography (radial free-breathing MR elastography) sequence (Online Supplementary Material 1). The motion encoding gradients were placed along the z direction (G_z_), and the data for each k_y_ line (Cartesian) or spoke (radial) was acquired twice with the polarity of the motion encoding gradients swapped between the two repetition times (TR). The radial free-breathing MR elastography sequence acquired 402 spokes to satisfy the Nyquist sampling criteria for an image matrix of 256×256. Radial spokes were acquired with golden-angle ordering to improve robustness to motion [32]. The Cartesian breath-hold and the radial free-breathing MR elastography sequences were each acquired twice (scans 1 and 2; mean time interval of 20 min) in the same exam to evaluate agreement and repeatability. MR elastography sequence parameters were matched as closely as possible (Table 1). The mechanical wave amplitude was set at the discretion of the MR technologist to achieve diagnostically viable images. The mechanical wave amplitude was 30% for small, petite (BMI≤18 kg/m^2^) and/or younger children, 40% for children with normal BMI (18–25 kg/m^2^), and 50–60% for children with higher BMI (≥25 kg/m^2^). Radial and Cartesian sequences used matched mechanical wave amplitudes.

**Table 1:** Representative imaging parameters

	Cartesian BH MR elastography	Radial FB MR elastography
TE	21.3 ms	21.3 ms
TR	50 ms	50 ms
Flip angle	25 degrees	25 degrees
Breath hold (BH) time	22 s	N/A
Field of view (FOV)	350×285 mm^2^	350×350 mm^2^
Reconstructed in-plane resolution	1.4×1.4 mm^2^	1.4×1.4 mm^2^
Slice thickness	5 mm	5 mm
Number of slices	4	1
Slice gap	1 mm	N/A
Acquired matrix size	128×104	256×256
Reconstructed matrix size	256×208	256×256
Interpolation	On	Off
Radial views	N/A	402
BW	399 Hz/pixel	400 Hz/pixel
Fat saturation	Off	Off
PAT (GRAPPA)	2	Off
MEG frequency	60 Hz	60 Hz
Wave amplitude	30–60%	30–60%
MEG direction	slice	slice
Scan time per slice	22 s (1 BH)	2 min 43 s
Total scan time	2 min 27 sec^a^ (4 BH, 4 slices)	5 min 26 s (2 slices)^b^

Breath-hold gradient echo (GRE) magnetic resonance (MR) elastography scans were acquired with 2-D Cartesian sampling, whereas the free-breathing GRE MR elastography scans used golden-angle-ordered 2-D radial sampling. The imaging parameters used for the Cartesian breath-hold MR elastography and radial free-breathing MR elastography sequences were matched as closely as possible. The slice positions for breath-hold and free-breathing MR elastography were matched for the 2–3 chosen slices in each subject. Analysis included the matched slices. The mechanical wave amplitude was adjusted between 30% and 60% depending on the subjects’ body characteristics.

^a^Includes BH instructions and breaks, ^b^For one subject, three slices were acquired in ~9 mins

*BH* breath hold, *BW* readout bandwidth, *FB* free breathing, *GRAPPA* generalized auto-calibrating partial parallel acquisition, *MEG* motion encoding gradients, *N/A* not applicable, *PAT* parallel imaging, *TE* echo time, *TR* repetition time

The Cartesian breath-hold MR elastography sequence acquired four slices (four breath-holds) during end-expiration, while the prototype radial free-breathing MR elastography sequence acquired a single slice per free-breathing scan. For Cartesian breath-hold-MR elastography, the duration for each breath-hold was 22 s, and the acquisition of four slices (4 breath-holds) took 2 min 27 s (including breath-hold instructions and breaks). The acquisition time for radial free-breathing MR elastography (single slice) was 2 min 43 s. Depending on the timing considerations and subject comfort, 2 or 3 slices were acquired with radial free-breathing MR elastography. These two or three slices were chosen to match the anatomy and the slice positions with the largest measurable liver region of interest (ROI) size in the Cartesian breath-hold MR elastography stiffness maps, as determined by the numerical confidence masks [33]. These matched slices from subjects were used for subsequent analyses. To facilitate comparisons, the order of the first acquisitions (i.e. scan 1) were always fixed as Cartesian breath-hold MR elastography followed by radial free-breathing MR elastography to align the free-breathing MR elastography slices to slices of interest in the breath-hold MR elastography scan. Once the positions of the radial free-breathing MR elastography slices were set according to the Cartesian breath-hold MR elastography during the first set of scans, the order of the second acquisitions (i.e. scan 2) of Cartesian breath-hold MR elastography and radial free-breathing MR elastography were randomized.

All the Cartesian breath-hold MR elastography and radial free-breathing MR elastography images and stiffness maps were reconstructed using the scanner software, with the same vendor-provided MR elastography processing algorithm. All data and statistical analyses were performed in MATLAB (2018b; MathWorks, Natick, MA).

### Hepatic stiffness measurement

**Fig. 1:**
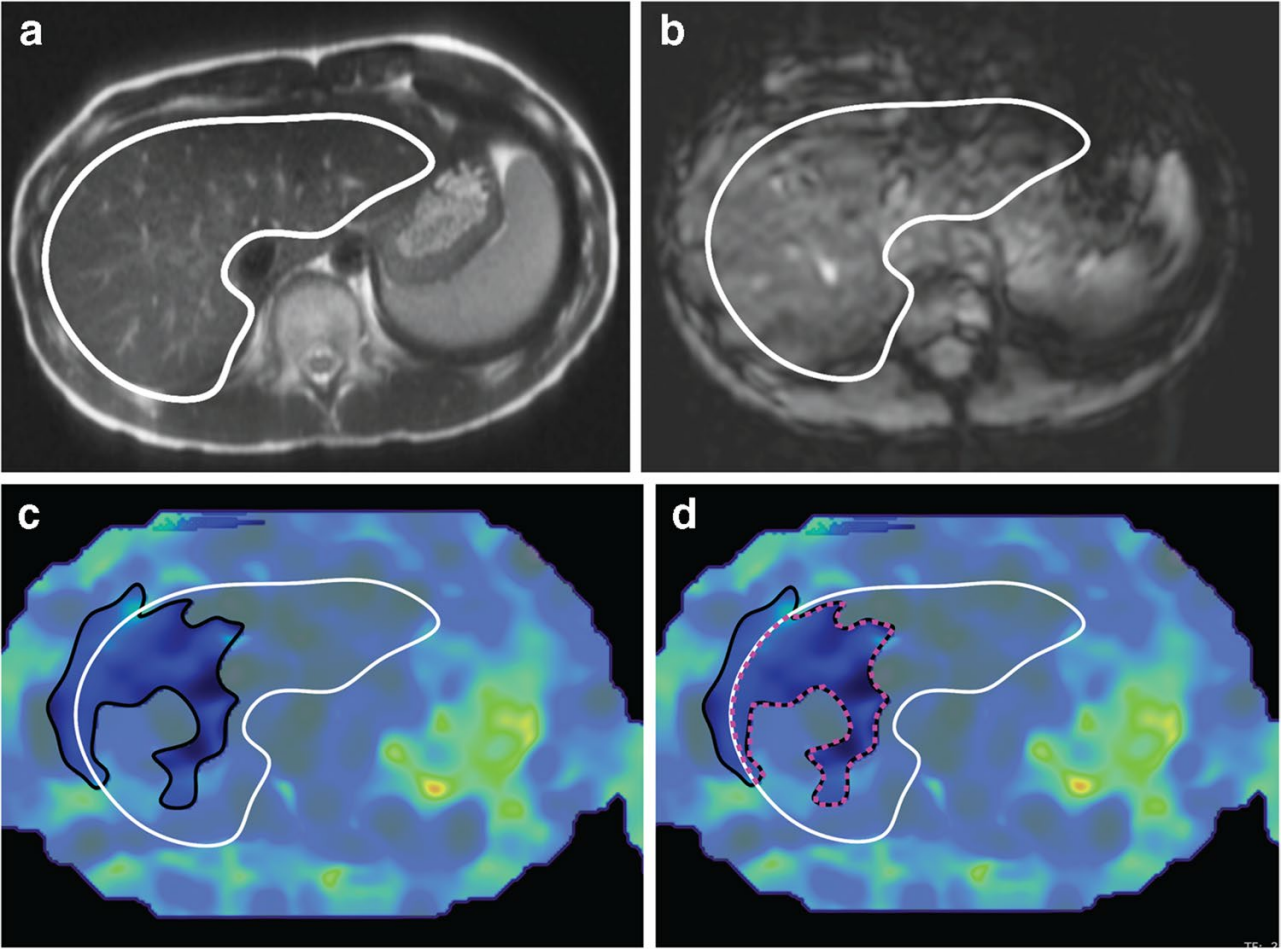
The steps for determining the region of interest for measuring hepatic stiffness in an 11-year-old girl with a body mass index of 19.2 kg/m^2^. The example shown is for Cartesian breath-hold magnetic resonance (MR) elastography. The liver was annotated using the axial T2-HASTE (half-Fourier acquisition single-shot turbo spin-echo) (**a**) and the MR elastography **(b)** axial magnitude image (*white contour*). The annotations were performed by a trained researcher and then adjusted/confirmed by a radiologist. The liver contour from the first step was copied to the corresponding hepatic stiffness map **(c)**. In addition, the region with at least 90% numerical confidence was automatically determined from the numerical confidence mask using custom software and overlaid on top of the hepatic stiffness map (*black contour*). The region of interest for measuring hepatic stiffness **(d)** was automatically determined as the intersection between the 90% confidence and liver masks, shown by the pink dashed lines

Hepatic stiffness was measured from ROIs inside areas of the liver with ≥90% confidence [19] using a custom MATLAB software tool. First, the liver was contoured in each subject and slice by a trained researcher (S.G.K., 3 years of experience) on the MR elastography magnitude images, using the T_2_-weighted half-Fourier acquisition single-shot turbo spin-echo (HASTE) images as an anatomical reference. The liver was contoured using recommendations by the Radiological Society of North America (RSNA) Quantitative Imaging Biomarkers Alliance (QIBA) Profile for Magnetic Resonance Elastography of the Liver, exclusion of the portal vein and heart as well as contouring predominantly the right lobe [12]. The liver contours were adjusted/confirmed by an experienced pediatric radiologist (S.G., >10 years of experience). Next, the software tool used the numerical confidence masks from the MR elastography processing algorithm to automatically create a mask that included areas with ≥90% confidence, as 90% confidence was used in a previous study conducting hepatic stiffness measurements in children [19]. In each subject, the ROI for measuring hepatic stiffness was then automatically determined from the intersection of the liver contour (black lines), and the 90% confidence mask (white lines), shown in Fig. 1 with the pink dashed lines. Stiffness values were reported as mean±standard deviation (SD) for each slice. The hepatic stiffness values were also reported for each subject and scan using the weighted mean of hepatic stiffness from all slices, which considers the normalized measurable liver area and the hepatic stiffness values across all slices. This way of measuring hepatic stiffness minimized variability originating from reader preferences in the ROI placement.

### Normalized measurable liver area

To assess the technical quality of the Cartesian breath-hold MR elastography and radial free-breathing MR elastography hepatic stiffness maps, we assessed and compared the measurable liver area based on numerical confidence masks [33, 34]. For this purpose, we defined the normalized measurable liver area for each subject as the measurable liver ROI area (see the hepatic stiffness measurement section) normalized to the total liver area in all slices included for analysis (in %) [29, 34]. Each sequence and repeated scan (i.e. scans 1 and 2) were analyzed separately for each subject. The normalized liver area from each breath-hold scan (scans 1 or 2) was compared with the corresponding free-breathing scan using median ± interquartile range (IQR) across subjects.

### Agreement between breath-hold and free-breathing MR elastography hepatic stiffness

Agreement in the mean hepatic stiffness measurements over matching slice positions for each subject and scan between Cartesian breath-hold MR elastography and radial free-breathing MR elastography was evaluated using Lin’s concordance correlation coefficient (CCC) [35] with 95% confidence intervals (CI). In addition, Bland-Altman analysis was performed for each repeated scan to compare the Cartesian and radial techniques in terms of their mean difference and 95% limits of agreement (LoA) defined as ±1.96 × *SD*, where *SD* denotes the standard deviation of the differences [36].

### Repeatability of breath-hold and free-breathing MR elastography hepatic stiffness

Intra-session repeatability in hepatic stiffness measurements was assessed for the repeated Cartesian breath-hold MR elastography and radial free-breathing MR elastography scans using the within-subject coefficient of variation [12, 37, 38]. Within-subject coefficient of variation was calculated using the formulation in the RSNA QIBA Profile for Magnetic Resonance Elastography of the Liver:$\sqrt{\sum_{i=1}^N\left( wS{D}_i^2/{Y}_i^2\right)/N\ }$where *N* is the total number of subjects, $wSD_i$ is the within-subject SD of the hepatic stiffness measurements of subject *i*, and *Y*_*i*_ is the mean of the hepatic stiffness measurements from two repeated scans of subject *i* [12]. Additionally, single-measure two-way mixed-model intraclass correlation coefficients (ICC) with 95% CI [39–41] were used to assess pairwise hepatic stiffness measurements from two repeated scans of Cartesian breath-hold MR elastography and radial free-breathing-MR elastography.

## Results

### Subject characteristics

We enrolled 14 healthy children (6 males, age [median, interquartile range]=[11.4, 2.8] years) and 9 children with chronic liver disease (6 males, age = [15.4, 3.1] years). Demographic information is reported in Table 2. All healthy children completed the MR elastography scans. Out of nine children with liver disease, seven completed the MR elastography scans. One 8-year-old boy could not follow breath-hold instructions. One 15-year-old boy with a BMI of 38.6 kg/m^2^ was not comfortable inside the scanner due to his body size and complained about the MR elastography paddle. Out of seven subjects with liver disease who completed the study, one had a liver biopsy and the results confirmed fibrosis (Table 2).

**Table 2:** Demographic information for healthy children and children with chronic liver disease

	Healthy subjects	Subjects with liver disease
Total number of subjects	14	9
Total number of subjects who completed the study	14	7
Age (median, IQR)	[11.4, 2.8]	[15.8, 2.6]
BMI (median, IQR)	[19.1, 4.2]	[33.1, 13.6]
Sex	8 F, 6 M	3 F, 4 M
Types of liver disease	N/A	57% (4) NAFLD/NASH^a^ 14% (1) biliary atresia 14% (1) cystic fibrosis 14% (1) autoimmune hepatitis/cirrhosis
Liver biopsy	0% (0)	14% (1)

The age and body mass index (BMI) of the subjects are reported as a median and interquartile range (IQR). Types of liver diseases, liver biopsy, and subject race and ethnicity are reported as percentages. Out of nine subjects with liver disease, seven completed the scans, and their data are reported here

^a^The subject with NASH had a biopsy 6 months before the magnetic resonance elastography scan, which confirmed grade 3 steatosis and stage 3 fibrosis (F3)

*BMI* body mass index, *F* female, *M* male, *N/A* not applicable, *NAFLD* nonalcoholic fatty liver disease. *NASH* nonalcoholic steatohepatitis

### Representative MR elastography experimental results

**Fig. 2:**
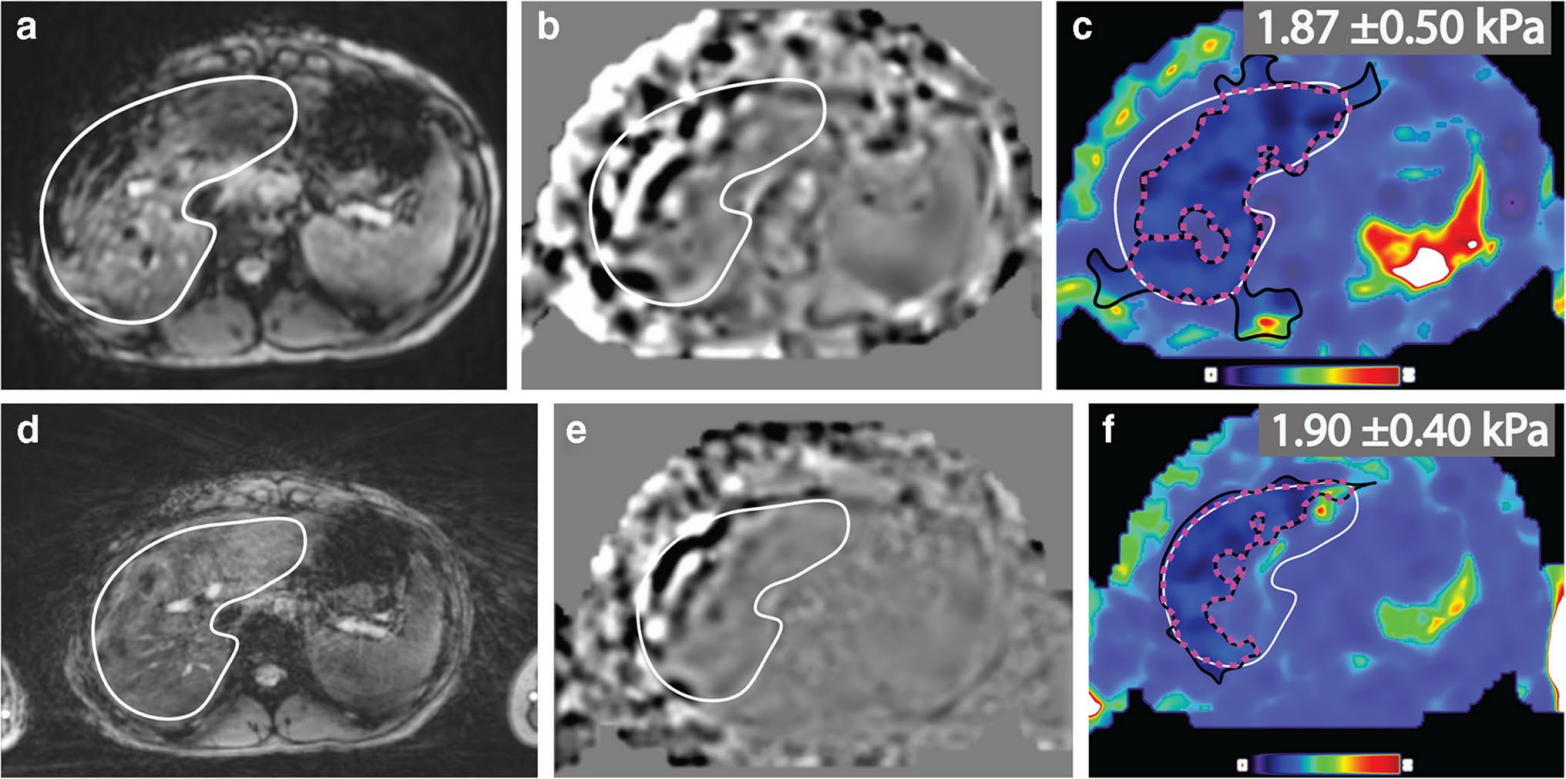
Representative axial magnetic resonance (MR) elastography with Cartesian breath-hold (**a-c**) and radial free-breathing (**d-f**) sequences. Magnitude images (**a, d**), wave images (**b, e**) and stiffness maps (**c, f**) with 90% confidence masks (*black contours*) and liver contours (*white contours*) are shown for a healthy 17-year-old girl with body mass index of 21.8 kg/m^2^. The hepatic stiffness was measured inside the region indicated by the pink dashed lines. The measured hepatic stiffness values from Cartesian breath-hold MR elastography and radial free-breathing MR elastography were consistent with fibrosis stage 0. For the scan shown here (scan 1), the normalized measurable liver area in the stiffness maps for radial free-breathing MR elastography (51.6%) was smaller than that of Cartesian breath-hold MR elastography (84.1%)

**Fig. 3:**
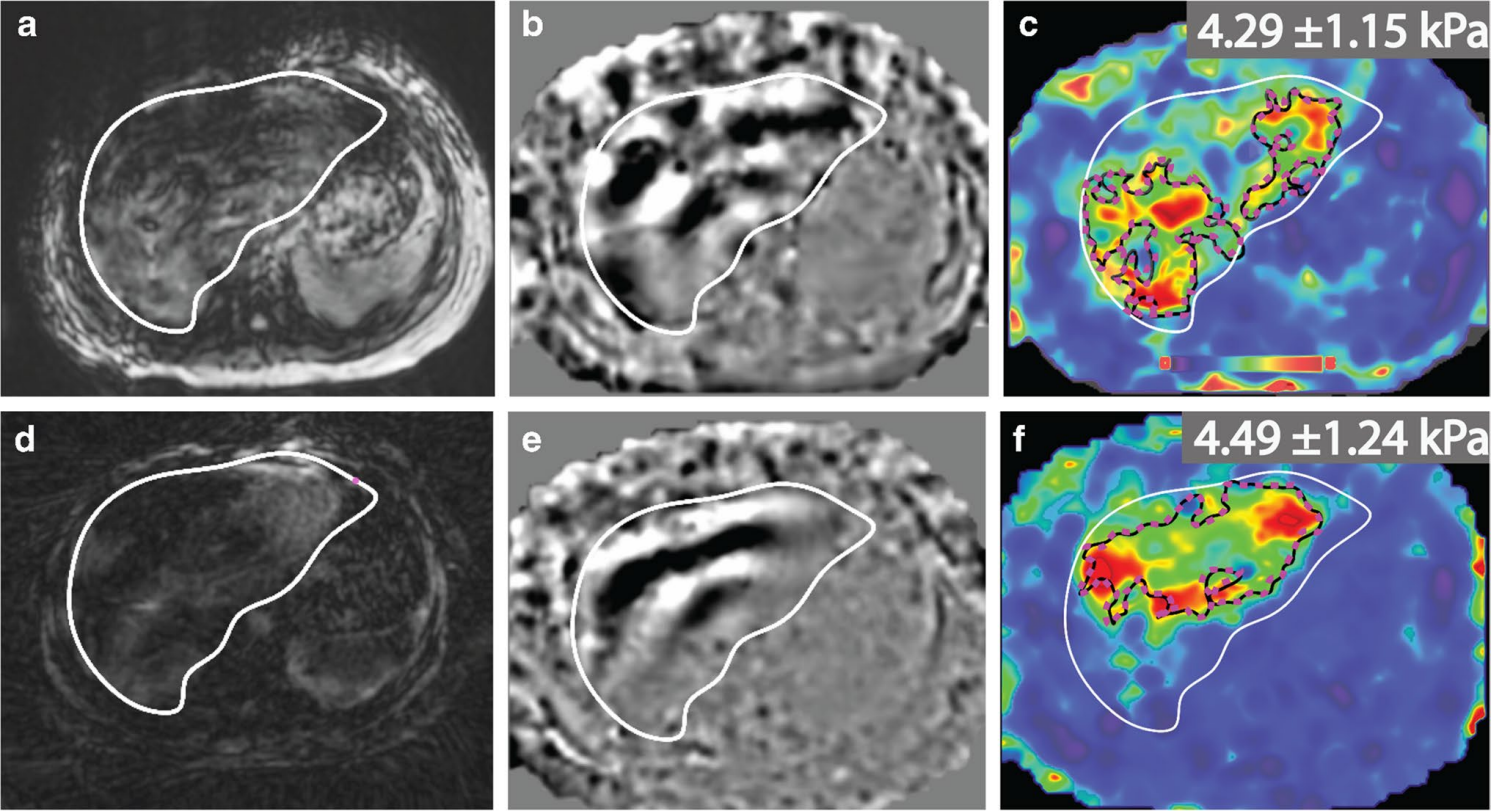
Representative axial magnetic resonance (MR) elastography with Cartesian breath-hold (**a-c**) and radial free-breathing (**d-f**) sequences. Magnitude images (**a, d**), wave images (**b, e**) and stiffness maps (**c, f**) with 90% confidence masks (*black contours*) and liver contours (*white contours*) are shown for a subject with biopsy-confirmed nonalcoholic steatohepatitis (12-year-old girl with body mass index of 27.9 kg/m^2^). The hepatic stiffness was measured inside the region indicated by the pink dashed lines. The stiffness values from Cartesian breath-hold MR elastography and radial free-breathing MR elastography were consistent with the biopsy results for this subject (stage 3 fibrosis). For the scan shown here (scan 1), the normalized measurable liver area in the stiffness maps for radial free-breathing MR elastography (59.5%) was larger than that of Cartesian breath-hold MR elastography (50.7%)

Representative examples of the MR elastography magnitude images, wave images and stiffness maps with 90% confidence masks and liver contours are shown in Figs. 2 and 3. The mean hepatic stiffness values measured by Cartesian breath-hold MR elastography and radial free-breathing MR elastography were consistent with each other. Table 3 summarizes the metrics and corresponding results for normalized measurable liver area (%), agreement of hepatic stiffness between Cartesian and radial techniques, as well as the repeatability of each technique.

**Table 3:** Summary of the metrics and results for normalized measurable liver area (%), agreement, and repeatability of hepatic stiffness values

Normalized measurable liver area (%)		
	Cartesian BH MR elastography	Radial FB MR elastography
Median (± IQR), scan 1	62.6% (± 26.4%)	44.1% (± 39.6%)
Median (± IQR), scan 2	60.3% (± 21.8%)	43.9% (± 44.2%)
Agreement of hepatic stiffness		
Mean difference, scan 1	0.03 kPa	
Mean difference, scan 2	−0.01 kPa	
LoA, scan 1	0.75 kPa	
LoA, scan 2	0.61 kPa	
CCC, scan 1	0.89 (95% CI: [0.78, 0.94])	
CCC, scan 2	0.94 (95% CI: [0.86, 0.97])	
Repeatability of hepatic stiffness		
	Cartesian BH MR elastography	Radial FB MR elastography
wCV	1.9%	3.4%
ICC	0.93 (95% CI: [0.84, 0.97])	0.92 (95% CI: 0.82, 0.97)

*BH* breath-holding, *CCC* Lin’s concordance correlation coefficient, *CI* confidence interval, *FB* free-breathing, *ICC* intraclass correlation coefficient, *LoA* limits of agreement, *wCV* within-subject coefficient of variation

### Normalized measurable liver area

**Fig. 4:**
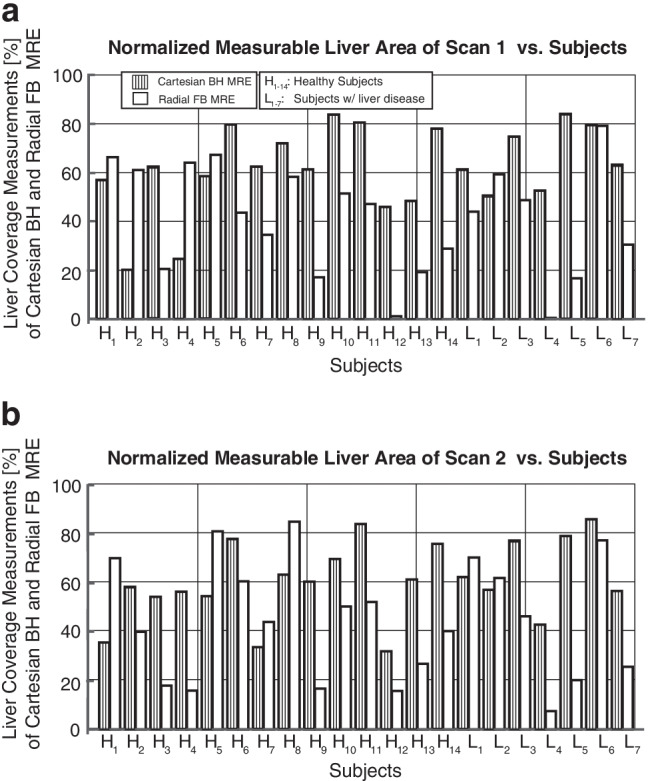
Normalized measurable liver area (in %) from two repeated scans for Cartesian breath-hold magnetic resonance (MR) elastography and radial free-breathing MR elastography. Scan 1 (**a**). Scan 2 (**b**). *BH*: breath-hold, *FB*: free-breathing, *MRE*: magnetic resonance elastography

Figure 4 shows the normalized measurable liver area values (in %) for each subject and two repeated Cartesian breath-hold MR elastography and radial free-breathing MR elastography scans. Similar results were obtained for Cartesian breath-hold MR elastography and radial free-breathing MR elastography, except for one healthy subject (H_12_) and one subject with liver disease (L_4_). The [median, IQR] of the normalized measurable liver area for scans 1 and 2 were [62.6%, 26.4%] and [60.3%, 21.8%] for Cartesian breath-hold MR elastography, and [44.1%, 39.6%] and [43.9%, 44.2%] for radial free-breathing MR elastography, respectively (Table 3).

### Agreement between breath-hold and free-breathing MR elastography hepatic stiffness

**Fig. 5:**
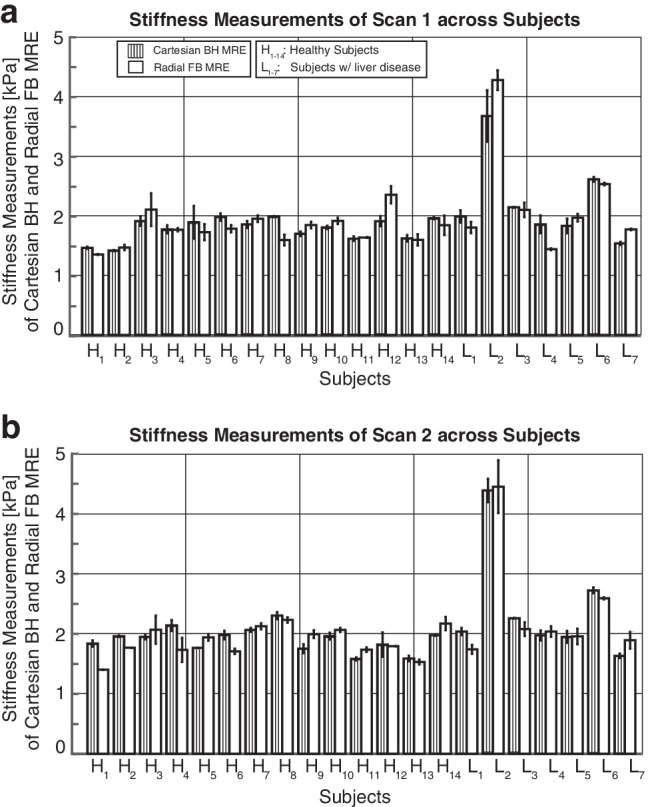
Cartesian breath-hold magnetic resonance (MR) elastography and radial free-breathing MR elastography had comparable mean hepatic stiffness measurements for two repeated scans. Scan 1 (**a**). Scan 2 **(b)**. The weighted mean stiffness measurements from each slice were calculated for each subject and sequence and used to compute the within-technique mean and the within-subject standard deviation (black bars). *H*_*1–14*_ healthy subjects, *L*_*1–7*_ subjects with liver disease, *BH*: breath-hold, *FB*: free-breathing, *MRE*: magnetic resonance elastography

**Fig. 6:**
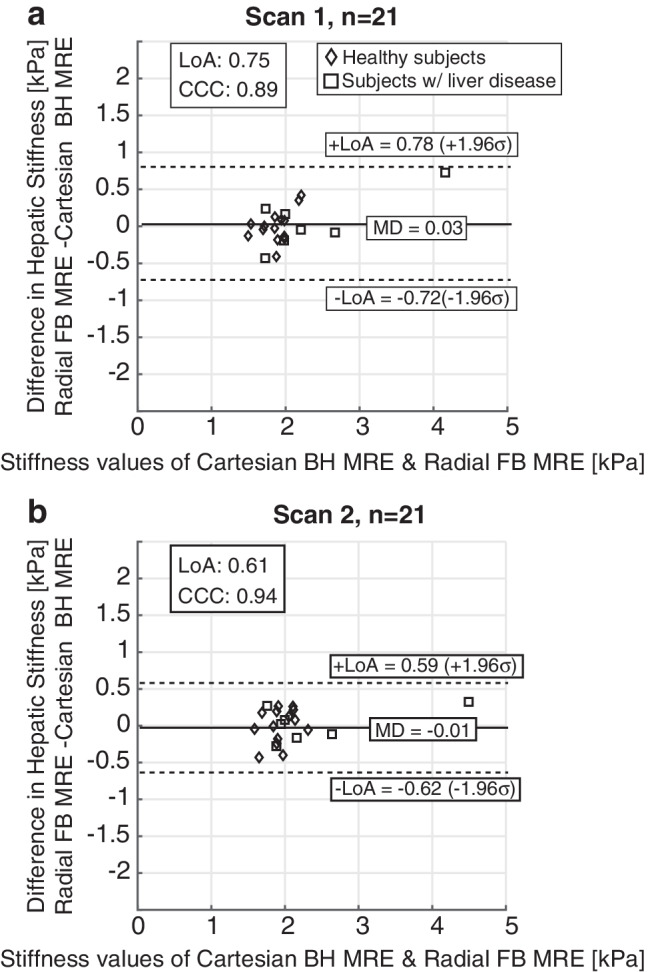
Agreement analysis using Lin’s concordance correlation coefficient (CCC) and Bland-Altman plots with 95% limits of agreement (LoA) and mean difference (MD) are shown for Cartesian breath-hold magnetic resonance (MR) elastography and radial free-breathing MR elastography for scan 1 (**a**) and scan 2 (**b**). High CCC (CCC=0.89 and 0.94 for scans 1 and 2, respectively) and small mean difference values (0.03 kPa for scan 1 and −0.01 kPa for scan 2) indicate that the hepatic stiffness values from Cartesian breath-hold MR elastography and radial free-breathing MR elastography are in agreement with each other for both scans. *BH*: breath-hold, *FB*: free-breathing, *MRE*: magnetic resonance elastography

Figure 5 compares the mean and SD of hepatic stiffness measurements for scans 1 and 2 separately for Cartesian breath-hold MR elastography and radial free-breathing MR elastography. Mean hepatic stiffness measurements for scans 1 and 2 for Cartesian breath-hold MR elastography and radial free-breathing MR elastography were comparable, with slight differences in SD values. Analysis results using CCC and Bland-Altman plots with mean difference and 95% LoA are shown for Cartesian breath-hold MR elastography and radial free-breathing MR elastography separately for scans 1 and 2 (Fig. 6). For scans 1 and 2, CCC=0.89 (95% CI: [0.78, 0.94]) and 0.94 (95% CI: [0.86, 0.97]) indicate excellent agreement in mean hepatic stiffness measurements between Cartesian breath-hold MR elastography and radial free-breathing MR elastography (Table 3). For scan 1, Bland-Altman analysis results were mean difference of 0.03 kPa and LoA of [−0.72, 0.78] kPa between Cartesian breath-hold MR elastography and radial free-breathing MR elastography. The Bland-Altman analysis results for scan 2 were consistent with scan 1, yielding mean difference of −0.01 kPa and LoA of [−0.62, 0.59] kPa.

### Repeatability of breath-hold and free-breathing MR elastography hepatic stiffness

The within-subject coefficient of variation values for Cartesian breath-hold MR elastography and radial free-breathing MR elastography were 1.9% and 3.4%, respectively, indicating similar repeatability (Table 3). Likewise, Cartesian breath-hold MR elastography and radial free-breathing MR elastography yielded similar ICC values of 0.93 (95% CI: [0.84, 0.97]) and 0.92 (95% CI: [0.82, 0.97]), respectively.

## Discussion

In this pilot study of healthy children and children with chronic liver disease, we compared a prototype radial free-breathing MR elastography sequence to a conventional 2-D Cartesian breath-hold MR elastography sequence at 3 T. We investigated normalized measurable liver area, agreement and repeatability for measuring hepatic stiffness. The novelty of this study was to investigate the technical performance of radial free-breathing MR elastography in children. To examine the degree of correlation and the strength of agreement between measurements from two imaging techniques, we used Lin’s concordance correlation coefficient and Bland-Altman analysis as recommended by the RSNA QIBA [37, 38]. Our results show that radial free-breathing MR elastography produced hepatic stiffness values that agreed with Cartesian breath-hold MR elastography, with a high Lin’s concordance correlation coefficient and low mean difference.

This work showed that Cartesian breath-hold MR elastography and radial free-breathing MR elastography yielded similar within-subject coefficient of variation and ICC, demonstrating comparable repeatability. According to the Profile for Magnetic Resonance Elastography of the Liver published by RSNA QIBA, within-subject coefficient of variation is a fundamental technical performance metric [12, 13]. Current clinical breath-hold MR elastography techniques achieve a within-subject coefficient of variation of 7%, which indicates that a true change in hepatic stiffness of 19% can be detected with 95% confidence [12, 13]. In this study, we found that Cartesian breath-hold MR elastography and radial free-breathing MR elastography within-subject coefficient of variation values were both less than 7%, indicating consistent and acceptable repeatability.

Overall, the radial free-breathing MR elastography sequences produced slightly smaller normalized measurable liver area compared to Cartesian breath-hold MR elastography for both scans 1 and 2. In particular, the healthy child H_12_ had smaller measurable liver area size for radial free-breathing MR elastography in both repeated scans due to increased bulk movement in addition to respiratory motion throughout the entire duration of radial free-breathing MR elastography. In subjects with liver disease, radial free-breathing MR elastography tended to yield lower normalized measurable liver area. One obese subject (L_4,_ BMI of 43.0 kg/m^2^) experienced discomfort inside the scanner during the radial free-breathing MR elastography scan. As a result, the measurable liver area was reduced. Despite these differences, the measurable liver area in radial free-breathing MR elastography hepatic stiffness maps included the most representative region (especially the right lobe of the liver), and measurements were repeatable and consistent with breath-hold MR elastography. For reference, a recent study in 264 adults with known or suspected liver disease reported the mean normalized measurable area as 31±20% for a Cartesian breath-hold GRE MR elastography sequence [42]. In our study, Cartesian breath-hold and radial free-breathing MR elastography techniques both yielded larger median normalized liver areas than the reported average from this previous study, suggesting that both techniques in our study achieved appropriate technical quality.

The hepatic stiffness values we measured in children are consistent with previous reports. One previous study using Cartesian breath-hold MR elastography in 81 healthy children (mean±SD age: 12.6±2.6 years) reported that the distribution of hepatic stiffness was 2.45±0.35 kPa [43]. Another study in children and young adults described slightly greater hepatic stiffness measurement values of 2.77±0.63 kPa for all patients with F0 [14]. The hepatic stiffness values we measured in healthy children using Cartesian breath-hold MR elastography and radial free-breathing MR elastography were lower, but consistent with this range. Another previous multisite and prospective study enrolled 90 children with NAFLD (age of 13.2±2.4 years) who underwent Cartesian breath-hold MR elastography [19]. Twelve of these children had fibrosis (stage ≥F2). A cutoff range between F0 versus F1-F4 was determined as 2.69 to 2.78 kPa. The stiffness values from healthy subjects in our study were consistently below this reported cutoff threshold.

For children, Cartesian breath-hold MR elastography may be challenging. Many children cannot breath-hold and follow instructions, particularly young children, children with chronic diseases and obese children [19, 21, 22]. New Cartesian and radial free-breathing MR elastography techniques may help overcome these limitations [8, 25, 26, 29]. To assess agreement between radial free-breathing MR elastography and Cartesian breath-hold MR elastography in this study, we enrolled children who were expected to be capable of breath-holding. Once validated in larger cohorts, the proposed radial free-breathing MR elastography technique could be used in children who cannot perform breath-holding. This is important because many chronic liver diseases affect toddlers and young children.

During MRI exams, breath-hold scans are typically acquired at either end-inspiration or end-expiration. A study analyzing the effects of different breath-hold states on the hepatic stiffness measurements in healthy adults showed that stiffness values changed significantly depending on whether the subject was scanned during a breath-hold at end-inspiration or end-expiration, for both 2-D Cartesian GRE and SE-EPI breath-hold MR elastography [44]. We demonstrated close agreement in hepatic stiffness between radial free-breathing MR elastography and Cartesian breath-hold MR elastography in this study. This is likely because Cartesian breath-hold MR elastography was acquired during end-expiration, and the current radial free-breathing MR elastography calculates hepatic stiffness from the entire free-breathing acquisition, resulting in an average of all motion states with end-expiration being the most common state. Future work could investigate the value of resolving hepatic stiffness over different motion states.

Only a few studies have investigated free-breathing MR elastography in adults [25, 26, 29]. There are even fewer studies in children [8]. Most studies used Cartesian free-breathing MR elastography at 1.5 T in adults [25, 26] and children [8]. However, Cartesian trajectory-based sequences are sensitive to motion-induced artifacts. Navigator triggering is a potential solution to this issue. One study in adults showed that navigator-triggered Cartesian free-breathing MR elastography and Cartesian breath-hold MR elastography had high concordance (CCC=0.716) [26]. However, children often have variable breathing patterns, and navigator triggering may lead to longer or variable scan times while still resulting in images with motion artifacts [22]. In contrast to Cartesian sampling, radial trajectories have greater inherent motion robustness for free-breathing acquisition [27, 28]. A previous study assessed the initial feasibility of a radial free-breathing MR elastography technique with self-navigated motion compensation at 1.5 T in two healthy subjects and two patients with liver disease, with ages ranging from 14 to 52 years [29]. Our current radial free-breathing MR elastography method also relies on the inherent motion robustness of golden-angle ordered 2-D radial sampling, but does not perform additional motion compensation. This may already provide sufficient motion robustness for free-breathing acquisition during relatively regular breathing patterns. For more irregular breathing patterns, such as variable and heavy breathing, self-navigated motion compensation [28, 29] could be incorporated to enhance the quantification accuracy and repeatability of radial free-breathing MR elastography.

There are limitations to our study. First, this is a single-site study of a small group of children. Further studies in larger cohorts with a wider range of hepatic stiffness are required to assess the diagnostic performance of free-breathing MR elastography. Second, only one subject had a biopsy. This is not surprising considering biopsies are invasive procedures that can be associated with bleeding and infections and are thus often avoided in children. As a result, a correlation analysis between biopsy and hepatic stiffness measurements was not possible. Third, due to the relatively long acquisition time of the current radial free-breathing MR elastography technique (2 min 43 s per slice), only two or three slices were acquired because of time constraints and patient comfort. Rapid [45], fractional [46] MR elastography with simultaneous-multi-slice imaging [47] and/or parallel imaging techniques for radial acquisition [48, 49] may be investigated to reduce radial free-breathing MR elastography scan time to facilitate clinical translation. The 2-D Cartesian breath-held GRE MR elastography sequence in clinical liver imaging protocols at our institution is setup as a 22 s/slice acquisition with in-plane resolution of 1.4 mm×1.4 mm and parallel imaging factor of 2 (Table 1). To reduce the breath-hold duration for liver MR elastography, other studies have utilized either 2-D Cartesian GRE with lower in-plane resolution [19], 2-D Cartesian spin-echo echo-planar imaging (2-D SE-EPI) [34, 50], or 2-D Cartesian GRE with rapid motion encoding and fractional encoding (2-D rapid fractional GRE) [51]. To have a fair comparison in terms of imaging parameters, this pilot study matched the 2-D Cartesian breath-held GRE and the 2-D radial free-breathing GRE MR elastography sequences as closely as possible. In future developments, we plan to reduce the scan time of radial free-breathing MR elastography and compare its agreement and repeatability with breath-held MR elastography methods such as 2-D Cartesian SE-EPI and 2-D Cartesian rapid fractional GRE. Fourth, the slice locations from breath-hold MR elastography and free-breathing MR elastography might not be perfectly matched due to intersequence motion and changes in the liver tissue during breath-hold and free-breathing. To address this problem, we carefully determined slice positions and liver contours based on anatomical context for corresponding Cartesian breath-hold MR elastography and radial free-breathing MR elastography scans to analyze hepatic stiffness. Lastly, the radial free-breathing MR elastography method is based on a prototype sequence that is not commercially available yet. We will continue to improve this prototype sequence in future work and move toward clinical translation.

## Conclusion

Hepatic stiffness measurements from 2-D radial free-breathing MR elastography were repeatable and in agreement with 2-D Cartesian breath-held MR elastography in a small cohort of healthy children and children with chronic liver disease at 3 T. These results show that radial free-breathing MR elastography is a promising technique to quantify hepatic stiffness in children when breath-holding is challenging or not possible. Further investigation is warranted.

## Supplementary Information

ESM 1 (DOCX 47 kb)
