# Supplementary material for: Free-breathing radial magnetic resonance elastography of the liver in children at 3 T: a pilot study
Source: Pediatr Radiol. 2022 Apr 2;52(7):1314–25. doi: 10.1007/s00247-022-05297-8 (PMC9192470; doi:10.1007/s00247-022-05297-8)

# Online Supplementary Material

# Online Supplementary Material 1 Sequence diagram of gradient-echo based radial free-breathing MR elastography. The motion encoding gradients (MEG) were placed along the z gradient (G_z_.) direction. The polarities of the MEGs were swapped every other repetition time (TR), where every TR is synchronized with 3 cycles of motion induced by the mechanical wave.

Flow compensating gradients are not shown for simplicity. RF: Radiofrequency pulse. TE: echo time. 𝜃 : Radiofrequency pulse flip angle.


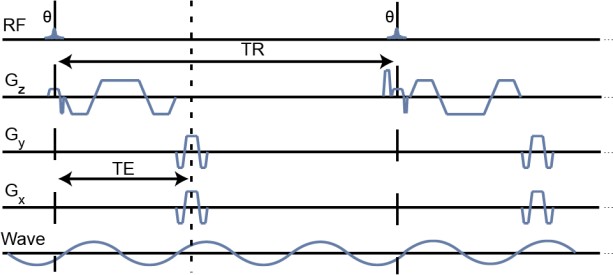

Supplement: Supplementary file 1 — (DOCX 47 kb) [file 247_2022_5297_MOESM1_ESM.docx]
